# Benign Lymphoid Hyperplasia Presenting as Bilateral Scleral Nodules

**DOI:** 10.1155/2015/179609

**Published:** 2015-09-01

**Authors:** Ricardo J. Cumba, Rene Vazquez-Botet

**Affiliations:** Ophthalmology Department, Pediatric Service, University of Puerto Rico, Medical Science Campus, P.O. Box 365067, San Juan, PR 00936, USA

## Abstract

*Purpose*. To report a case of transient lymphoid hyperplasia presenting as bilateral nodular scleral mass in a young male patient. *Design*. Observational case report. *Methods*. Chart review. Causes of scleritis were considered and excluded based on detailed history, physical examination, and laboratory investigations. *Results*. Excisional biopsy of scleral lesions indicated lymphoid tissue. Immunohistochemical studies revealed a polyclonal population of T and B cells consistent with a benign reactive process. *Conclusions*. Chronic exposure of the ocular adnexa to many allergens and irritants may lead to activation of the inflammatory cascade. In severely allergic patients activation may be exponential and elicit an immune-mediated response resulting in a transient lymphoid reactive process.

## 1. Introduction

Ocular adnexal lymphoproliferative lesions are a spectrum of diseases that includes reactive lymphoid hyperplasia, atypical lymphoid hyperplasia, and lymphoma [1]. Most of these lesions are located to the conjunctiva, eyelid, orbit, and lacrimal glands [2]. Diagnosing such lesions is challenging due to considerable overlap between malignant and reactive processes [3]. We present an unusual case of transient lymphoid hyperplasia presenting as bilateral scleral nodular mass in a young male patient.

## 2. Case Report

**Figure 1:**
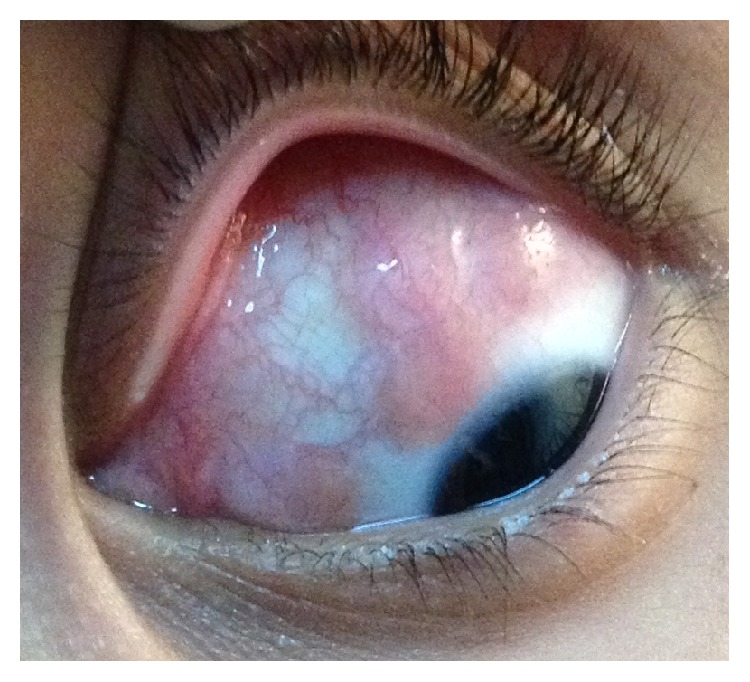
Left eye showing nodular scleral lesions in front of the rectus muscles at time of presentation.

**Figure 2:**
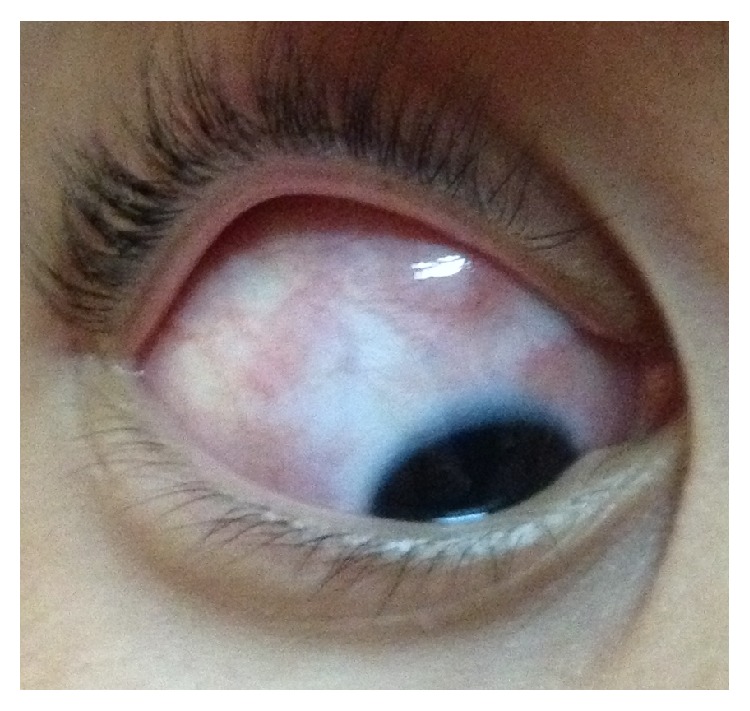
Right eye showing less prominent superior scleral lesions and redness at sites of old nodular lesions two months after presentation.

**Figure 3:**
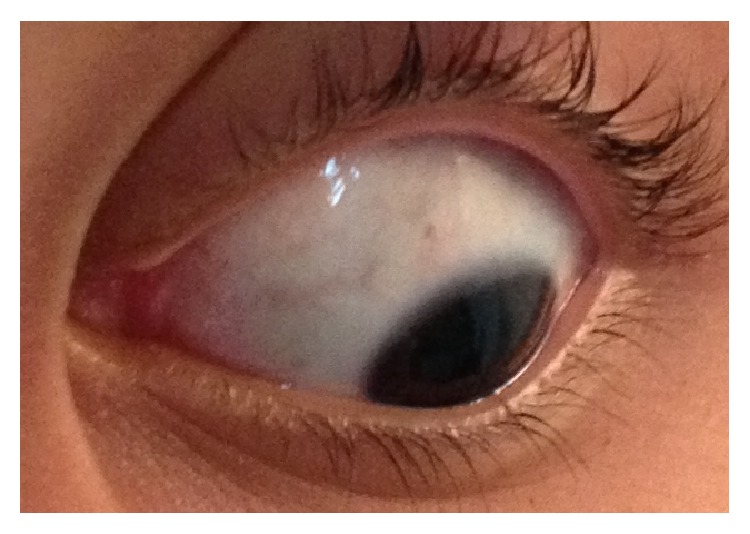
Left eye showing complete resolution of scleral lesions four months after presentation.

A 10-year-old male presents with a three-month history of painless scleral lesion in both eyes. He had been treated with topical steroids and 60 mg of prednisone PO for suspected nodular episcleritis/scleritis by two ophthalmologists. Patient was brought for a second opinion on whether prednisone should be restarted. His past medical history was unremarkable, except for allergies, and there was no recent travel. On examination his visual acuity was 20/20 in both eyes. Slit lamp exam showed diffuse nodular scleral infiltrates in front of the insertion of the extraocular muscles in both eyes with no extraocular movement compromise (Figure 1). Fundus exam was unremarkable. Systemic examination was normal with no evidence of lymphadenopathy. Investigations requested included CBC, ESR, CRP, CXR, serum ACE and lysozyme, VDRL, FTA-Abs, U/A, ANA, ANCA, Lyme titers, and MRI of head and orbit with contrast which were all within normal limits. Prednisone was not started, and biopsy of the area in front of the left superior rectus and left lateral rectus was performed. Biopsy was performed under general anesthesia, with limbal base approach opening of the conjunctiva. There were no complications during the procedure and minimal bleeding was noted. Patient was able to resume his usual activities the next day after the procedure and did not complain of any ocular pain during postoperative period. He was treated with Maxitrol drops three times a day for two weeks. The H&E section revealed a nodular small lymphocytic infiltrate containing histiocytes in a dense fibrous background. The lymphocytes did not show significant cytologic atypia and no well-formed germinal centers or granulomas were identified. These results suggested either prominent primary B-cell follicles or involvement of a low grade B-cell lymphoma. Immunohistochemical studies showed that lymphocytes were mostly B cells positive for CD20, weak CD23, and BCL-2 but were negative for CD5, CD10, CD43, BCL-1, and BCL-6. The CD3 positive small T cells were mostly present in between or in the periphery of the B-cell nodules, which were morphologically unremarkable. Staining of CD21 highlighted follicular dendritic meshworks associated with the nodular areas, and MUM-1 positive plasma cells appeared to be polytypic. This polyclonal population of T and B cells was consistent with a benign reactive process (Table 1). Patient was evaluated by pediatric-oncologist who also ordered serum protein electrophoresis, which was normal, and did not find any evidence suggesting malignancy. Therefore, he attributed this episode to his history of allergies. Patient was observed and lesions slowly regressed in the next four months with no further prednisone treatment (Figures 2 and 3).

## 3. Discussion

The ocular adnexa are a common site for the development of benign lymphoid hyperplasia. These lesions exist in a spectrum with atypical lymphoid hyperplasia and lymphomas. With no pathognomonic symptoms, differentiating benign from malignant processes is challenging [4]. The pathogenesis of ocular adnexal lymphoproliferative lesions has eluded us; however, some cases seem to have association with autoimmune diseases [5, 6]. In our case, the patient had no prior history of any autoimmune diseases but did suffer from recurrent severe episodes of allergic rhinitis.

It is theorized that an allergic reaction may be the first of 3 developmental phases involved during the entire course of inflammatory responses. Inflammation is a series of complex immunologic and physiologic responses of tissues to irritants and allergens. There is evidence of a direct association between inflammation and the development of tumor-like lesions in lymphoid tissues [7, 8]. The first phase during the immune response of inflammation involves IgE-mast cell sensitization and degranulation. This is followed by an intermediate phase, a desensitization phenomenon, and loss of mast cell function and neovascularization. Finally, there comes a chronic response where there is induction of massive lymphoid hyperplasia, follicular formation with germinal centers, increased swollen goblet cells, extensive epithelial thickening and thinning, and angiogenesis [7, 8]. This inflammatory response is also known to promote changes in adjacent tissues. It is possible that some of these inflammatory mediators produced by the conjunctiva may have spread through the superficial vessels of the episclera and the interscleral vascular plexus located just posterior to the limbus eliciting a lymphoid proliferation in the sclera of our patient.

Scleral biopsy is not routinely performed on patients with anterior scleral nodules, especially if scleritis is suspected. Most of these biopsies will yield nonspecific results. However, in atypical cases that do not respond to treatment, scleral biopsy is indicated to aid in the diagnosis of tumoral, inflammatory, or infectious masquerade syndromes. Although a simple procedure, there is always the risk of eye perforation, infection, and scleral thinning at site of biopsy that may require tissue reinforcement grafts [9].

We describe a case of transient lymphoid hyperplasia that presented as a diffuse, nonpainful, scleral nodular infiltrate resembling a nodular episcleritis/scleritis. With no symptoms or clinical criteria to differentiate malignant processes, tissue biopsy is needed to aid in diagnosis. Findings of polyclonal population of lymphocytes on immunohistochemistry are consistent with a benign reactive process. Repeated stimulation of tissue by allergens in chronic allergic patients may elicit a severe antigen-antibody reaction, which then promotes benign lymphoid proliferation [10, 11]. We believe this to be the pathophysiology in our patient. However, there was no evidence of eosinophilia on CBC or biopsy, and serum protein electrophoresis at time of presentation did not show IgE elevation.

To date, after two years of follow-up, the patient has had an entirely benign clinical course with no evidence of a lymphoproliferative disorder. To the best of our knowledge, and, after extensive literature search, this is the only reported case of an ocular adnexal lymphoproliferative process in which the site of lymphoid infiltration is the sclera instead of the conjunctiva, orbit, eyelid, or lacrimal gland.

## Figures and Tables

**Table 1:** Immunohistochemistry analysis.

Marker	Result	Description
CD3 (PS1)	T cells positive	Pan T cell, epsilon subunit of the CD3 T cell receptor complex
CD5 (4C7)	T cells positive	Pan T cell antigen, mature B cell subset, and thymic carcinoma
CD10	Negative	Follicle center B cells, CALLA (B and T ALL)
CD20	B cells positive	Pan B cell antigen (L26)
CD21	Follicular dendritic cells positive	C3d and EBV receptor, mature B cells, and follicular dendritic cells
CD23 (MHM6)	B cells positive	Low affinity IgE receptor, mature B cells, and CLL/SLL
CD43	T cells positive	T cells, B cell subset, myeloid cells, and histiocytes (Leu22)
CD68	Histiocytes/macrophages positive	Macrophages and myeloid cells (KP1)
PAX-5 (BSAP)	B cells positive	B cell and Hodgkin's lymphoma
BCL-1	Negative	Mantle cell lymphoma (Cyclin D1, PRAD-1)
BCL-2 IHC	Lymphocytes positive	Antiapoptosis protein, follicular lymphoma, and B cell subset
BCL-6	Negative	Follicle center B cells
MUM-1	Plasma cells positive	Plasma cells postgerminal center B cells, activated T cells
Kappa (Px)	Polytypic in plasma cells	Kappa immunoglobulin light chain, B cells, and plasma cells
Lambda (Px)	Polytypic in plasma cells	Lambda immunoglobulin light chain, B cells, and plasma cells
Ki-67 (MIB-1)	1–4%	Cell proliferation marker (MIB-1)

## References

[1] Liesegang T. J. (1993). Ocular adnexal lymphoproliferative lesions. *Mayo Clinic Proceedings*.

[2] Coupland S. E. (2004). Lymphoproliferative lesions of the ocular adnexa. Differential diagnostic guidelines. *Ophthalmologe*.

[3] Beykin G., Pe'er J., Amir G., Frenkel S. (2014). Paediatric and adolescent elevated conjunctival lesions in the plical area: lymphoma or reactive lymphoid hyperplasia?. *British Journal of Ophthalmology*.

[4] Alkatan H. M., Alaraj A., El-khani A., Al-Sheikh O. (2013). Ocular adnexal lymphoproliferative disorders in an ophthalmic referral center in Saudi Arabia. *Saudi Journal of Ophthalmology*.

[5] Kubota T., Moritani S. (2007). High incidence of autoimmune disease in Japanese patients with ocular adnexal reactive lymphoid hyperplasia. *The American Journal of Ophthalmology*.

[6] Stacy R. C., Jakobiec F. A., Schoenfield L., Singh A. D. (2010). Unifocal and multifocal reactive lymphoid hyperplasia vs follicular lymphoma of the ocular adnexa. *American Journal of Ophthalmology*.

[7] Khatami M. (2005). Developmental phases of inflammation-induced massive lymphoid hyperplasia and extensive changes in epithelium in an experimental model of allergy: implications for a direct link between inflammation and carcinogenesis. *American Journal of Therapeutics*.

[8] Khatami M. (2014). Chronic inflammation: synergistic interactions of recruiting macrophages (TAMs) and eosinophils (EoS) with host mast cells (MCs) and tumorigenesis in CALTs. M-CSF, suitable biomarker for cancer diagnosis. *Cancers*.

[9] de la Maza M. S., Tauber J., Foster C. S. (2012). *The Sclera*.

[10] Khatami M., Donnelly J. J., Haldar J. P., Wei Z.-G., Rockey J. H. (1989). Massive follicular lymphoid hyperplasia in experimental allergic conjunctivitis: local antibody production. *Archives of Ophthalmology*.

[11] Dagklis A., Ponzoni M., Govi S. (2012). Immunoglobulin gene repertoire in ocular adnexal lymphomas: hints on the nature of the antigenic stimulation. *Leukemia*.

